# Exploratory Measurements of Singly Substituted and Clumped Isotopologues in Iodate Using Electrospray Ionization Mass Spectrometry

**DOI:** 10.1002/rcm.70161

**Published:** 2026-09-08

**Authors:** Jamie Lucarelli, Amanda R. Bubas, Richard M Cox, Sophie B. Lehmann, Aradhna Tripati, Mark Engelmann, Elise R. Conte

**Affiliations:** ^1^ Department of Earth, Planetary, and Space Sciences UCLA Los Angeles California USA; ^2^ Pacific Northwest National Laboratory Richland Washington USA

## Abstract

**Rationale:**

Clumped isotope geochemistry can reveal the temperatures and chemical pathways at which molecules form, but it remains unexplored for most inorganic systems. Iodate (IO_3_^−^) is central to ocean and atmospheric iodine cycling and to tracking radioactive iodine from nuclear activity. Here, we report the first exploratory investigation into the feasibility of measuring bulk and clumped isotopologues in iodate using electrospray ionization mass spectrometry (ESI‐MS).

**Methods:**

Sodium iodate was dissolved in either deionized water or 97% H_2_^18^O. Two sample sets systematically varied the H_2_^18^O fraction of the spray solvent from 0% to 97%. Samples were infused into a Thermo Scientific TSQ Quantum Access MAX triple‐quadrupole mass spectrometer with an atmospheric pressure ionization source operated in negative‐ion scan mode. Spectra were acquired over *m/z* 110–190 and averaged across 25 scans.

**Results:**

The singly substituted isotopologue (^127^I^16^O_2_^18^O^−^, *m/z* 177), clumped isotopologues (^127^I^16^O^18^O_2_^−^, *m/z* 179; ^127^I^18^O_3_^−^, *m/z* 181), and isotopologue distributions were resolved in IO_3_^−^. Relative isotopologue abundances shifted monotonically with solvent ^18^O content; ^127^I^18^O_3_^−^ increased from 0% to 56% across the enrichment range. In the natural abundance sample, measured isotopologue abundances matched the values predicted for a stochastic (random) isotope distribution, confirming that the instrument is sampling the natural isotopologue distribution without significant bias. We also report isotopologue distributions for IO^−^ and IO_2_^−^, which exhibit isotopic trends similar to those observed for IO_3_^−^.

**Conclusions:**

These first measurements of isotopologues in IO_3_^−^ establish ESI‐MS as an accessible platform for isotopic analysis of inorganic oxyanions under enriched conditions. We discuss the thermodynamic basis for equilibrium clumping in IO_3_^−^, the analytical requirements for extending these enriched sample measurements to natural abundance clumped isotope thermometry, and the potential applications of this system in Earth and nuclear sciences.

## 1 Introduction

The study of clumped isotopes—molecules or ions containing two or more rare, heavy stable isotopes (e.g., ^13^C^18^O^16^O or ^15^N_2_)—has emerged as an important branch of isotope geochemistry over the past two decades. Unlike traditional bulk isotope measurements, which quantify the ratio of heavy to light isotopes relative to an international standard, clumped isotope analysis measures the degree to which two or more heavy isotopes co‐occur within the same molecule relative to a stochastic (random) isotope distribution [1, 2]. This nonrandom ordering arises from quantum mechanical effects: bonds involving multiple heavy isotopes have lower vibrational zero‐point energies (ZPEs) and are therefore thermodynamically stabilized at low temperatures [3]. Consequently, the degree of equilibrium isotopic clumping is temperature dependent, with greater enrichment of clumped species at lower temperatures [1, 3]. Clumped isotope measurements thus provide powerful, compositionally independent tools for reconstructing mineral and fluid formation temperatures and for distinguishing equilibrium from kinetic isotopic processes.

Historically, clumped isotope geochemistry was developed for gas‐phase molecules, primarily CO_2_ liberated from carbonate minerals by phosphoric acid digestion. Foundational work by Eiler and Schauble established the Δ_47_ thermometer based on the abundance of mass‐47 CO_2_ (^13^C^18^O^16^O), which records carbonate and CO_2_ formation temperatures [2, 4]. Over the subsequent two decades, advances in isotope ratio mass spectrometry (IRMS) and isotope ratio laser spectroscopy (IRLS) expanded the field to include additional gas‐phase systems such as H_2_, O_2_, N_2_, and CH_4_, enabling discrimination among formation processes and source environments. More recently, the introduction of Orbitrap mass spectrometry enabled direct measurements of intact solution‐phase ions, including sulfate (SO_4_^2−^) and nitrate (NO_3_^−^), without requiring chemical conversion to a gaseous derivative and without disturbing the original bonding environment [5, 6]. Table 1 summarizes the molecules, primary clumped isotopologues, applications, and instrumentation used across the field to date.

**TABLE 1:** Overview of experimentally measured clumped isotopologue systems.

Phase	Molecule/ion	Primary clumped isotopologue(s)	Applications	Primary instrument(s)	Citation(s)
Gas	CO_2_	^13^C^18^O^16^O; ^12^C^18^O_2_	Palaeothermometry Metabolic CO_2_ Provenance Reaction kinetics	IRMS (Thermo MAT 253, Nu Perspective, Thermo 253 Plus); IRLS (Aerodyne TILDAS)	Ghosh et al. [1]; Eiler [2]; Fiebig et al. [7]; Lucarelli et al. [8]; Yanay et al. [9]
Gas	CH_4_	^13^CH_3_D; ^12^CH_2_D_2_	Thermometry Microbial vs. thermogenic gas discrimination	IRMS (Thermo 253 Ultra, Nu Panorama); IRLS (Aerodyne TILDAS)	Stolper et al. [10]; Wang et al. [11]; Haghnegahdar et al. [12]
Gas	O_2_	^18^O_2_	Ozone chemistry Biological productivity	IRMS (Thermo MAT 253)	Yeung et al. [13]
Gas	N_2_	^15^N_2_	Atmospheric mixing N‐fixation tracers	IRMS (Thermo 253 Ultra, Nu Panorama)	Yeung et al. [14]; Yan et al. [15]
Gas	N_2_O	^15^N_2_^16^O; ^14^N^15^N^18^O	N_2_O source attribution (e.g., fertilizer vs. soil)	IRLS (Aerodyne QCLAS); HR‐IRMS (Thermo 253 Ultra)	Magyar et al. [16]; Kantnerová et al. [17]
Gas	H_2_	D_2_	High‐temperature gas–rock reactions Hydrothermal systems	IRMS (Thermo 253 Ultra)	Popa et al. [18]; Mangenot et al. [19]
Gas	C_2_H_6_	^13^C_2_H_6_; ^13^C^12^CH_5_D	Natural gas maturity Formation temperature	IRMS (Thermo 253 Ultra; Thermo MAT 253)	Clog et al. [20]; Taguchi et al. [21]
Liquid	SO_4_^2−^	^34^S^18^O^16^O_3_^2−^	Tracing bacterial sulfate reduction	ESI‐Orbitrap (Thermo Q Exactive HF Orbitrap)	Neubauer et al. [5]
Liquid	NO_3_^−^	^15^ N ^18^ O ^16^ O_2_^−^ ; ^14^ N ^18^ O_2_^16^ O ^−^	Atmospheric oxidation Source apportionment	ESI‐Orbitrap (Thermo Orbitrap Exploris)	Walters et al. [6]

Abbreviations: IRLS, infrared laser spectroscopy; IRMS, isotope ratio mass spectrometry; QCLAS, quantum cascade laser absorption spectroscopy; TILDAS, tunable infrared laser direct absorption spectroscopy.

Despite these advances, clumped isotope measurements remain absent from many inorganic systems. Iodate (IO_3_^−^) is a critical component of both the marine and atmospheric iodine cycles [22] and is thermodynamically favored as the dominant dissolved iodine species in the open ocean [23]. Yet the formation pathways of IO_3_^−^, whether driven by purely abiotic processes or by microbial mediation, remain difficult to constrain using bulk isotopes alone [23, 24]. Prior work has demonstrated that the oxygen atoms in IO_3_^−^ exchange with ambient water [25], suggesting that the oxygen isotope composition of IO_3_^−^ may preserve information about the conditions under which the molecule formed or equilibrated. While iodine isotope tracers (e.g., ^129^I/^127^I) have provided valuable constraints on the rates and relative contributions of biotic and abiotic pathways to iodine redox transformations in seawater [24, 26], no existing approach directly constrains the temperature or mechanistic signature of IO_3_^−^ formation from within the molecule itself. Bulk oxygen isotope (δ^18^O) and clumped isotope analysis of IO_3_^−^ could potentially distinguish rapid kinetic oxidation (e.g., photochemical or abiotic pathways) from slow, equilibrium‐driven oxygen exchange within the marine iodine cycle. These redox transformations are closely linked to primary productivity, ocean mixing, and atmospheric exchange, as iodine emitted from the ocean surface plays a disproportionate role in tropospheric ozone destruction and aerosol formation [27, 28]. Given the tight coupling between iodine speciation and ozone in the marine boundary layer [27], mass independent fractionation (Δ^17^O) of iodate could in principle serve as an analogous proxy for ozone‐driven oxidation in IO_3_^−^ formation.

In parallel, iodine is of critical importance in anthropogenic systems, particularly nuclear. Radionuclides of iodine are produced during nuclear fission and released through nuclear processes, resulting in widespread environmental distribution across marine, atmospheric, and terrestrial reservoirs [29, 30]. In these environments, iodine speciation exerts primary control on mobility, persistence, and bioavailability, with iodate generally favored under oxic conditions and iodide exhibiting enhanced mobility and reactivity [30, 31]. While isotopic measurements of ^129^I/^127^I ratios have been used for source apportionment and dispersal of anthropogenic iodine, these approaches have limited insight into the mechanisms and physicochemical conditions governing iodine redox transformations [30, 32].

In this study, we explore a new analytical pathway to study oxygen and clumped isotopes using electrospray ionization mass spectrometry (ESI‐MS), a widely available platform used for molecular characterization and quantification in environmental, pharmaceutical, and forensic applications [33]. The ability of ESI‐MS to isolate precursor ions and perform collision‐induced dissociation (CID) enables targeted analysis of specific isotopologues, offering a potentially accessible route for resolving both bulk and clumped isotope signatures in inorganic oxyanions.

We report an exploratory investigation into the feasibility of measuring singly substituted (I^16^O_2_^18^O^−^) and clumped isotopologues (^127^I^16^O^18^O_2_^−^; ^127^I^18^O_3_^−^) in IO_3_^−^ using ESI‐MS. We characterize the behavior of bulk and clumped isotopologue signals across a range of oxygen isotope enrichment levels. This work demonstrates that ESI‐MS provides an accessible platform for isotopic measurements in inorganic oxyanions under isotopically enriched conditions and lays the groundwork for future quantitative clumped isotope studies in this system.

## 2 Methods

### 2.1 Sample Preparation

Sodium iodate (NaIO_3_) was purchased from Sigma‐Aldrich (St. Louis, MO). Two separate NaIO_3_ stock solutions of were prepared. The first stock solution was prepared by dissolving ~10 mg of NaIO_3_ salt in 2 mL of in‐house deionized water with a natural oxygen isotope distribution (^16^O ≈ 99.76%, ^18^O ≈ 0.20%, ^17^O ≈ 0.04%). The second stock solution was prepared by dissolving ~10 mg NaIO_3_ salt in 2 mL of H_2_^18^O (97 atom % ^18^O, Sigma‐Aldrich). Two sets of spray solutions for ESI‐MS analysis were prepared by diluting 20 μL of the respective NaIO_3_ stock solution into 1 mL of LC–MS grade methanol (MeOH) and 1 mL of water according to the proportions listed in Table 2. This design produced two sample sets (Set 1: NaIO_3_ dissolved in deionized H_2_O; Set 2: NaIO_3_ dissolved in H_2_^18^O) spanning the full range from ~0% to ~97% H_2_^18^O in the spray solvent. Samples were analyzed ≥ 1 h after they were prepared, allowing for isotopic equilibration (*t* ≥ *t*_99_).

**TABLE 2:** Composition of spray solutions prepared for ESI‐MS analysis, where each sample also contained 1 mL of LC–MS grade MeOH.

Sample ID	μL NaIO_3_ in deionized H_2_O	μL H_2_O	μL 97% H_2_^18^O	μL NaIO_3_ in 97% H_2_^18^O
1‐0	20	1000	0	0
1‐10	20	800	200	0
1‐20	20	600	400	0
1‐30	20	400	600	0
1‐40	20	200	800	0
1‐50	20	0	1000	0
2‐0	0	1000	0	20
2‐10	0	800	200	20
2‐20	0	600	400	20
2‐30	0	400	600	20
2‐40	0	200	800	20
2‐50	0	0	1000	20

### 2.2 ESI‐MS Analysis

A Thermo Scientific TSQ Quantum Access MAX equipped with an atmospheric pressure ionization (API) source coupled to the HESI‐II probe (Thermo Scientific) was used for all analyses presented herein. Spray solutions were introduced directly into the ESI source by infusion using the incorporated syringe pump set to a flow rate of 10 μL min^−1^. ESI source conditions were as follows: spray voltage of −3000 V, N_2_ sheath gas pressure of 10 arbitrary units, capillary temperature of 250°C, capillary offset of −35 V, tube lens offset of −51 V, and skimmer offset of 40 V. Mass spectra were acquired in negative‐ion scan mode over the mass range of 110–190 *m/z*, and each reported spectrum represents the average of 25 scans at 1 s per scan. Spectra were collected after allowing the solution to infuse and spray in the source to stabilize, generally ~5 min. Each sample solution was measured once, and therefore, isotopic stability of the solutions and measurement reproducibility was not examined. Raw and background corrected intensities for all samples are available in Tables S1 and S2, respectively.

### 2.3 Calculations

#### 2.3.1 Stochastic Isotopologue Abundances

Stochastic isotopologue abundances and exact masses for IO^−^, IO_2_^−^, and IO_3_^−^ were calculated assuming a stochastic oxygen isotope distribution, monoisotopic iodine (^127^I), and the isotopic abundances tabulated by the National Institute of Standards and Technology (NIST). For example, the stochastic fractional abundance of the clumped isotopologue ^127^I^16^O^17^O^18^O^−^ was calculated using the multinomial expression of Yergey [34]:

(1) $$\begin{array}{c} \frac{n!}{n_{16}!n_{17}!n_{18}!}\times {\left(p_{16}\right)}^{n_{16}}\times {\left(p_{17}\right)}^{n_{17}}\times {\left(p_{18}\right)}^{n_{18}}\\ =\frac{3!}{1!1!1!}\left(0.99757\right)\left(0.00038\right)\left(0.00205\right)=4.66\times 10^{-6} \end{array}$$

where *n* is the total number of oxygen atoms; *n*_16_, *n*_17_, and *n*_18_ are the numbers of each oxygen isotope; and *p*_16_, *p*_17_, and *p*_18_ are the respective natural isotopic abundances.

#### 2.3.2 Relative Isotopologue Abundances From Ion Intensities

Relative isotopologue abundances were calculated by normalizing the measured ion intensity of each isotopologue to the summed intensity of all IO_3_^−^ isotopologue signals acquired under identical analytical conditions. For example, the relative abundance of I^18^O_3_^−^ (*m/z* 181) for each sample was calculated as follows:

(2) $$\frac{I_{181}}{\sum_{i=175}^{181}I_{i}}$$

where *I*_181_ is the measured ion intensity at *m/z* 181 and the summation is taken over all IO_3_^−^ isotopologue intensities. Similar calculations were performed for IO^−^ and IO_2_^−^.

#### 2.3.3 Temperature‐ and pH‐Dependent Oxygen Isotope Equilibration Times

Equilibration timescales for oxygen isotope exchange between IO_3_^−^ and water were calculated from the rate law reported by Anbar and Guttmann [25], who measured exchange rates in unbuffered aqueous solutions at 25°C over the pH range 4.5–10.65. The observed first‐order rate constant for whole‐molecule isotopic exchange is

(3) $$k^{\prime }=k_{H}\left[H^{+}\right]+k_{\mathrm{OH}}\left[{\mathrm{OH}}^{-}\right]$$

where *k*′ has units of s^−1^. Anbar and Guttmann [25] report specific rate constants *k* defined through *R*_ex_ = *k*(IO_3_^−^), where *R*_ex_ is the rate of oxygen‐atom exchange. Because each iodate has three exchangeable oxygens, the McKay relation gives *R*_ex_ ≈ 3 *k*′(IO_3_^−^) when water is in large excess, so their tabulated coefficients equal 3*k*′. The whole‐molecule first‐order exchange constants are therefore obtained by dividing by 3:

$$k_{H}=3.24\times 10^{4}/3=1.08\times 10^{4}\;L\;{\mathrm{mol}}^{-1}\;s^{-1}$$

and

$$k_{\mathrm{OH}}=1.35\times 10^{3}/3=4.50\times 10^{2}\;L\;{\mathrm{mol}}^{-1}\;s^{-1}.$$

Because Anbar and Guttmann [25] report measurements only at 25°C, rate constants for *k*_H_ and *k*_OH_ at other temperatures were obtained using the Arrhenius equation and their respective activation energies:

(4) $$k\left(T\right)=k\left(25^{{}^{\circ}}C\right)\times \exp\left[-E_{a}/R\times \left(1/T-1/T_{\mathrm{ref}}\right)\right]$$

where *T* is the target temperature, *T*_ref_ is 298.15 K, and *R* = 8.314 × 10^−3^ kJ mol^−1^ K^−1^. Activation enthalpies (Δ*H* ^‡^) were taken from von Felten et al. [35], who measured the temperature dependence of IO_3_^−^–H_2_O oxygen exchange between 5°C and 25°C: Δ*H* ^‡^_H_ = 61.5 kJ mol^−1^ (acid‐catalyzed pathway) and Δ*H* ^‡^_OH_ = 47.4 kJ mol^−1^ (base‐catalyzed pathway). Activation energies (*E*_a_) were obtained from the activation enthalpies by *E*_a_ = Δ*H* ^‡^ + RT. The hydroxide concentration at each temperature was calculated from the temperature‐dependent water dissociation constant *K*_w_ [25, 35]:

(5) $$\left[{\mathrm{OH}}^{-}\right]=K_{w}\left(T\right)/\left[H^{+}\right]$$

Values of p*K*_w_ were taken from standard thermodynamic tables (14.94 at 0°C, 14.53 at 10°C, 13.996 at 25°C, 13.26 at 50°C, 12.70 at 75°C, and 12.26 at 100°C), with linear interpolation between tabulated values.

The time required to reach 99% oxygen isotope equilibration (*t*_99_) was calculated from the integrated first‐order rate law:

(6) $$t_{99}=\ln\left(100\right)/k^{\prime }=4.605/k^{\prime }$$

This follows from setting the fractional approach to equilibrium *F*(*t*) = 1 − exp(−*k*′*t*) = 0.99 and solving for *t*.

## 3 Results

### 3.1 Iodate Isotopologue Distributions: Sample Set 1

**FIGURE 1:**
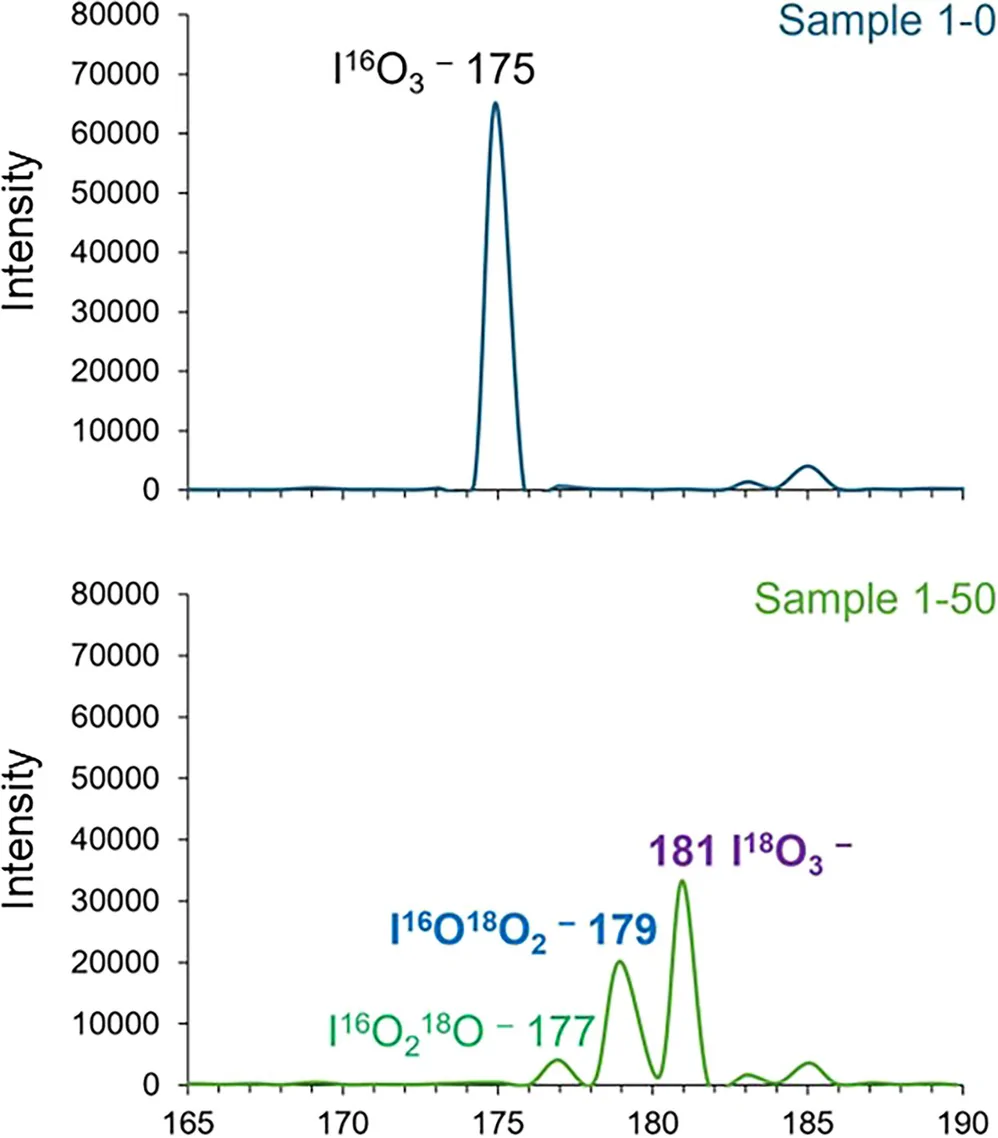
Negative‐ion ESI‐MS mass spectra for Samples 1‐0 and 1‐50, shown over the range *m/z* 165–190.

Representative ESI mass spectra from Sample Set 1 are shown in Figure 1. Sample 1‐0, prepared with water of natural oxygen isotopic composition, is dominated by peaks containing only ^16^O. The major peaks occur at *m/z* 127 (I^−^), 143 (IO^−^), 159 (IO_2_^−^), and 175 (IO_3_^−^), consistent with expectations for NaIO_3_ dissolved in deionized water. The measured relative abundance of *m/z* 175 in Sample 1‐0 (Table 3) is in close agreement with the predicted stochastic value of 99.27% (Table 4), confirming that the instrument is sampling the natural isotopologue distribution without significant bias.

**TABLE 3:** Relative percent abundances of all measured isotopologues for IO^−^, IO_2_^−^, and IO_3_^−^.

Sample	IO^−^ 143	IO^−^ 144	IO^−^ 145	IO_2_^−^ 159	IO_2_^−^ 160	IO_2_^−^ 161	IO_2_^−^ 162	IO_2_^−^ 163	IO_3_^−^ 175	IO_3_^−^ 176	IO_3_^−^ 177	IO_3_^−^ 178	IO_3_^−^ 179	IO_3_^−^ 180	IO_3_^−^ 181
1‐0	99.8	0.2	—	99.0	—	1.1	—	—	99.4	—	0.8	—	—	—	—
1‐10	79.5	1.3	19.2	74.3	1.4	20.5	0.6	3.2	56.3	0.8	34.6	0.4	7.1	0.1	0.7
1‐20	67.9	1.2	30.9	48.0	0.8	39.4	1.3	10.4	30.4	0.7	43.2	0.6	21.1	0.2	3.8
1‐30	48.1	5.6	46.3	31.6	1.3	45.9	0.9	20.3	12.8	1.0	35.2	1.3	36.4	0.2	13.2
1‐40	37.7	2.9	59.4	16.8	1.1	47.5	0.9	33.7	4.6	0.4	24.2	1.2	42.9	0.7	26.0
1‐50	20.5	2.5	77.0	3.7	—	31.7	1.6	63.0	0.5	—	6.7	0.6	33.9	2.1	56.1
2‐0	93.2	0.3	6.5	89.4	0.3	9.4	0.2	0.7	81.5	0.4	16.3	0.2	1.3	—	0.2
2‐10	77.3	0.2	22.5	65.5	1.2	29.7	0.3	3.3	46.3	0.7	40.2	0.4	11.1	0.1	1.2
2‐20	59.2	1.1	39.7	45.2	0.5	42.6	0.4	11.3	22.0	0.9	41.8	0.9	28.1	0.5	5.8
2‐30	52.9	1.3	45.8	38.9	0.5	43.4	0.4	16.7	17.8	1.1	38.6	0.8	32.8	0.3	8.5
2‐40	36.7	2.8	60.5	15.0	1.3	43.0	1.5	39.2	4.0	0.3	24.7	1.3	40.4	0.8	28.5
2‐50	21.8	1.7	76.4	5.8	0.5	30.6	1.7	61.5	1.2	0.4	9.2	1.1	34.7	1.3	52.2

**TABLE 4:** Stochastic relative abundances of naturally occurring IO^−^, IO_2_^−^, and IO_3_^−^ isotopologues.

Molecule	Isotopologue	Relative abundance (%)
IO^−^	^127^I^16^O^−^	99.757
	^127^I^18^O^−^	0.205
	^127^I^17^O^−^	0.038
IO_2_^−^	^127^I^16^O_2_^−^	99.515
	^127^I^16^O^18^O^−^	0.409
	^127^I^16^O^17^O^−^	0.076
	^127^I^18^O_2_^−^	4.203 × 10^−4^
	^127^I^17^O^18^O^−^	1.558 ×10^−4^
	^127^I^17^O_2_^−^	1.444 × 10^−5^
IO_3_^−^	^127^I^16^O_3_^−^	99.273
	^127^I^16^O_2_^18^O^−^	0.612
	^127^I^16^O_2_^17^O^−^	0.113
	^127^I^16^O^18^O_2_^−^	1.258 × 10^−3^
	^127^I^16^O^17^O^18^O^−^	4.663 ×10^−4^
	^127^I^16^O^17^O_2_^−^	4.321 × 10^−5^
	^127^I^18^O_3_^−^	8.615 × 10^−7^
	^127^I^17^O^18^O_2_^−^	4.791 × 10^−7^
	^127^I^17^O_2_^18^O^−^	8.881 × 10^−8^
	^127^I^17^O_3_^−^	5.487 × 10^−9^

**FIGURE 2:**
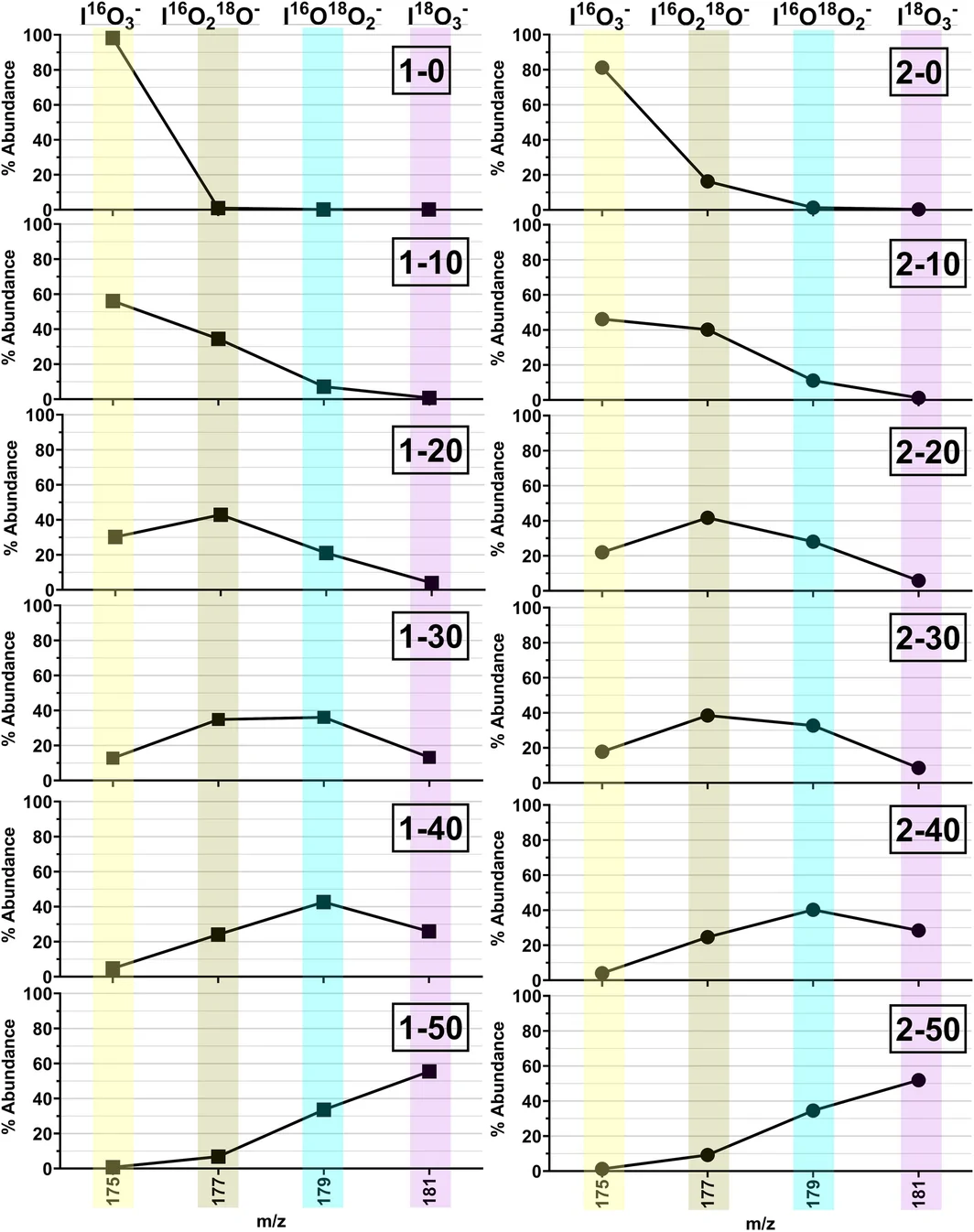
Relative percent abundances of IO_3_^−^ isotopologues measured in Sample Sets 1 (left panels) and 2 (right panels). In both sets, the ^18^O abundance increases from top to bottom, and the distributions shift toward ^18^O substituted isotopologues.

As the proportion of H_2_^18^O in the spray solution increases from Samples 1‐0 to 1‐50, systematic and monotonic changes in the isotopologue distribution are observed. Signals corresponding to ^18^O‐substituted species, including *m/z* 177 (I^16^O_2_^18^O^−^), 179 (I^16^O^18^O_2_^−^), and 181 (I^18^O_3_^−^), progressively increase in intensity. For example, the relative abundance of I^18^O_3_^−^ increases from 0.0% in Sample 1‐0 to 0.7%, 3.8%, 13.2%, 26.0%, and 56.1% in Samples 1‐10 to 1‐50, respectively (Table 3 and Figure 2). At intermediate compositions (Samples 1‐10 to 1‐40), all isotopologues are present at measurable intensities. When the proportion of H_2_^18^O exceeds that of H_2_^16^O (Samples 1‐30, 1‐40, and 1‐50), the ^18^O‐bearing isotopologues become the dominant species. Qualitatively similar trends are observed for the IO^−^ and IO_2_^−^ ion series.

In Sample 1‐50, the spectrum is dominated by *m/z* 179 (I^16^O^18^O_2_^−^) and *m/z* 181 (I^18^O_3_^−^), demonstrating that ^18^O from the solvent is incorporated into IO_3_^−^ and that the resulting isotopologue distributions reflect the bulk isotopic composition of the spray solution. The resolution of these peaks confirms that clumped isotopologues in IO_3_^−^ can be resolved by ESI‐MS when ^18^O is above natural abundance.

### 3.2 Iodate Isotopologue Distributions: Sample Set 2

In contrast to Sample Set 1, the NaIO_3_ in this series was initially dissolved in 97% H_2_^18^O, so that Sample 2‐50 represents the most ^18^O‐enriched condition. The spectrum of Sample 2‐50 was dominated by the clumped isotopologues at *m/z* 179 (I^16^O^18^O_2_^−^; 34.7%) and *m/z* 181 (I^18^O_3_^−^; 52.2%). Minor contributions from ^16^O‐bearing species are still present, reflecting oxygen contributions from the NaIO_3_ salt, water, and possibly from the MeOH cosolvent.

As the proportion of H_2_^16^O decreases from Samples 2‐0 to 2‐50, a systematic redistribution of isotopologues occurs (Figure 2). The relative abundance of I^16^O_3_^−^ at *m/z* 175 decreases progressively (81.5%, 46.3%, 22.0%, 17.8%, 4.0%, and 1.2%), while that of I^18^O_3_^−^ at *m/z* 181 increases correspondingly (0.2%, 1.2%, 5.8%, 8.5%, 28.5%, and 52.2%). These trends are consistent with those observed in Sample Set 1 and similarly apply to the IO^−^ and IO_2_^−^ ion series (Table 3).

Across both sample sets, singly substituted and clumped isotopologues are consistently resolved under ESI transmission conditions. Isotopologue relative abundances vary monotonically and systematically with the bulk oxygen isotopic composition of the spray solution (Figure 2 and Table 3).

## 4 Discussion

### 4.1 Thermodynamic Basis of Clumped Isotope Equilibria in IO_3_^−^

The thermodynamic basis of clumped isotope behavior lies in differences in vibrational ZPE among isotopologues [36]. Molecules containing multiple heavy isotopes exhibit lower vibrational frequencies and therefore lower ZPE, making these configurations thermodynamically favored at equilibrium relative to a stochastic isotope distribution [36, 37]. The extent of this thermodynamic preference diminishes with increasing temperature. For a harmonic oscillator, ZPE is related to the vibrational frequency *ν* by

(7) $$\mathrm{ZPE}=\frac{1}{2}\mathrm{h\nu}=\frac{1}{2}h\left(\frac{1}{2\pi }\sqrt{\frac{k}{\mu }}\right)$$

where *k* is the bond force constant, *h* is Planck's constant, and *μ* = *m*_1_ *m*_2_/(*m*_1_ + *m*_2_) is the reduced mass of the I–O atom pair. The equilibrium constant *K* for an isotope clumping reaction scales approximately as [2, 3, 37]

(8) $$\ln K\approx -\frac{\text{ΔZPE}}{\mathrm{RT}}$$

The reduced‐mass framework provides insight into the expected equilibrium sensitivity of IO_3_^−^. Substitution of ^18^O for ^16^O in the I–O bond produces a large relative change in reduced mass (Δ*μ* ≈ +1.56 amu; Δ*μ*/*μ* ≈ 0.11), yielding a significant reduction in vibrational frequency and enhanced thermodynamic preference for clumping relative to a stochastic distribution (Table 4). By comparison, the analogous CO_2_ clumped isotopologue (^13^C–^18^O relative to ^12^C–^16^O) gives Δ*μ* ≈ +0.69 amu and Δ*μ*/*μ* ≈ 0.10. The similar values suggest that IO_3_^−^ and CO_2_ possess comparable intrinsic reduced‐mass sensitivity to heavy isotope clumping, supporting IO_3_^−^ as a viable thermodynamic clumping system.

### 4.2 Equilibrium Clumping and Oxygen Isotope Exchange Kinetics in IO_3_^−^

Because iodine has only one stable isotope (^127^I), all stable isotopic variation in IO_3_^−^ arises from oxygen substitutions, which effectively eliminates cation‐related isotopic complexity and makes this an isotopically clean system for clumped isotope analysis. The dominant clumped isotopologues I^16^O^18^O_2_^−^ and I^18^O_3_^−^ reflect intramolecular isotope ordering governed by thermodynamic equilibrium. The large reduced‐mass shift associated with ^18^O substitution in the I–O bond produces significant ZPE reductions, enhancing thermodynamic ordering relative to the stochastic distribution (Table 4), particularly at low temperatures. These effects are analogous to equilibrium oxygen clumping in sulfate, where intramolecular isotope ordering is temperature‐dependent [5].

**FIGURE 3:**
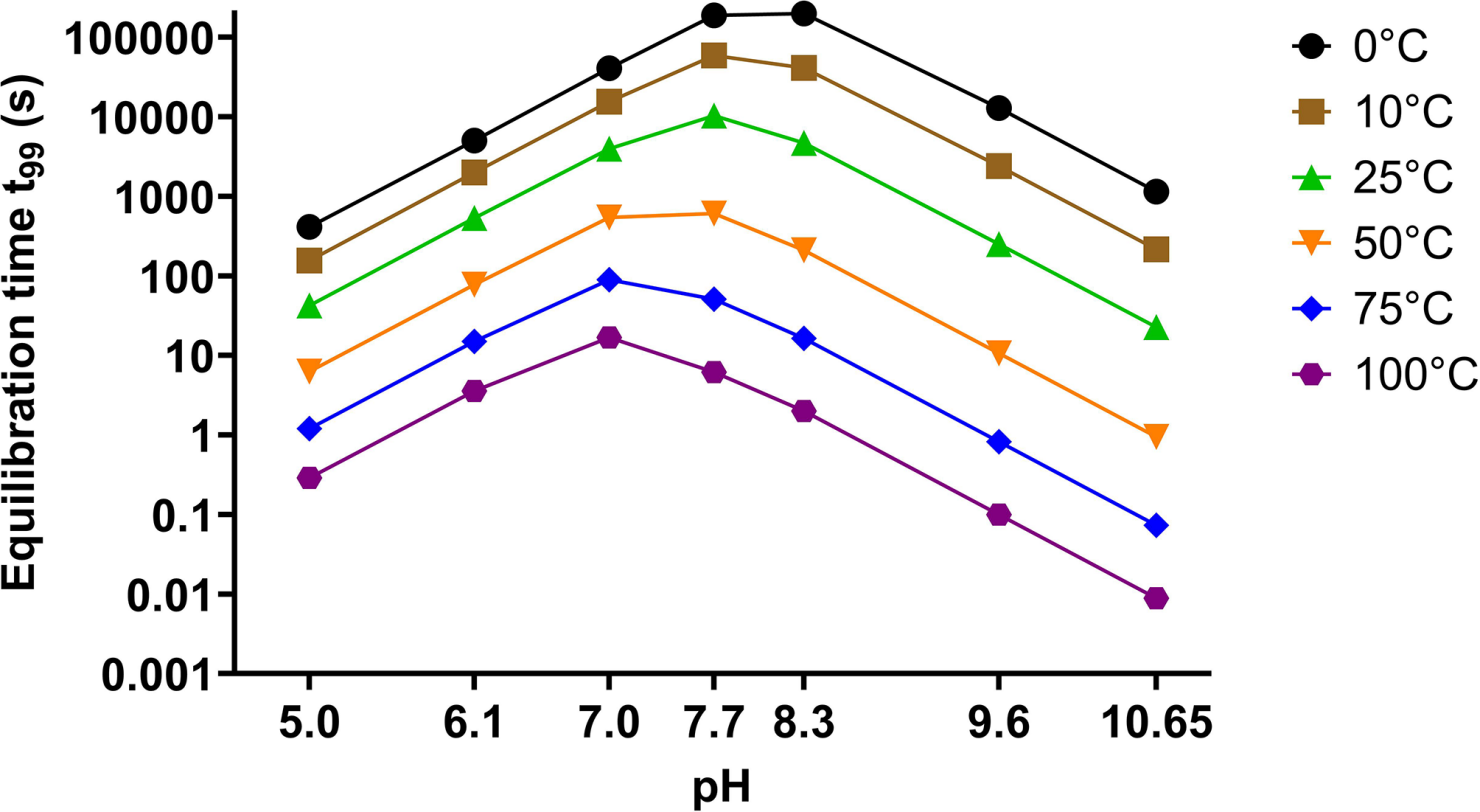
Calculated oxygen isotope equilibration time (*t*_99_) between IO_3_^−^ and water at varying solution pH and temperature.

The rate at which IO_3_^−^ achieves oxygen isotope equilibrium with water depends strongly on temperature and pH. Oxygen isotope exchange between IO_3_^−^ and H_2_O is both acid and base catalyzed, with a minimum rate near pH 7.7 [25]. Using the experimentally determined rate constants and Arrhenius scaling described in Section 2.3.3, the time required to achieve 99% oxygen isotope equilibration (*t*_99_) at pH 7 decreases from ~11 h at 0°C to ~9 min at 50°C (Figure 3 and Table S3). At 25°C, equilibration times range from ~43 s (at pH 5) to ~2.9 h (at pH 7.7) depending on pH. These short equilibration timescales, particularly at elevated temperatures, have important implications for the applicability of IO_3_^−^ as a thermometer and for sample handling protocols, as discussed in Section 4.3.

### 4.3 Translating ESI‐MS Measurements to Quantitative Isotope Ratio Analysis: Pathways and Challenges

The utility of ESI‐MS for clumped isotope analysis presents several challenges, and the ability to quantify absolute or relative concentrations of analytes using ESI‐MS alone has been debated [38, 39, 40]. It is important to note that the signal of the analyte of interest is influenced by the ionization source settings (e.g., spray voltage, gas flow rates, skimmer, and lens voltages). Additionally, the chemical environment of the sample (concentration, pH, and solvent matrix) is likely to influence spray stability and therefore ionization efficiency. Multiple reaction monitoring (MRM) offers a way to increase selectivity, which is achieved by selecting for a precursor ion of interest in the first quadrupole, fragmenting the precursor of interest by CID in the second quadrupole, and monitoring the intensity of specific, characteristic fragmentation products in the third quadrupole [41]. Other options to enhance quantitative capabilities include isotopic dilution or calibration curves produced using standard addition. Mass spectrometry can also be coupled with another analytical methodology such as gas chromatography (GC–MS) or liquid chromatography (LC–MS) to enhance quantitative capabilities, although traditionally, the primary objective is accurate compound identification and quantification rather than high precision isotope ratio measurement.

In contrast, clumped isotope analysis requires that measured ion intensities accurately reflect the relative abundances of clumped isotopologues compared to the corresponding unsubstituted species. Resolving these signals requires measurement precision on the order of 0.01‰–0.1‰ [2], utilizing careful control over detector linearity and signal stability. These requirements are challenging for IO_3_^−^ at natural isotopic abundance, where the key clumped isotopologues I^16^O^18^O_2_^−^ (*m/z* 179) and I^18^O_3_^−^ (*m/z* 181) represent 1.3 x 10^−3^% and ~8.6 × 10^−7^% of total IO_3_^−^, respectively (Table 4). Under isotopically enriched conditions, isotopologue abundance differences are orders of magnitude larger than natural and resolvable by ESI‐MS (Figures 1 and 2). For measurements at natural abundance targeting equilibrium clumped isotope thermometry, higher mass resolution and enhanced ion counting statistics, such as those provided by Orbitrap‐based systems, may be required.

Additionally, an important consideration for extending this framework to nuclear‐relevant systems is the potential role of ^129^I. While substitution of ^129^I for ^127^I produces a nominal mass shift of +2 amu (e.g., ^129^I^16^O_3_^−^ at *m/z* 177), these species are isobaric with ^18^O‐substituted ^127^I iodate isotopologues (e.g., ^127^I^16^O_2_^18^O^−^). At natural oxygen isotope abundances, the singly ^18^O‐substituted isotopologues occur at 0.6% relative abundance, meaning that detection of ^129^I‐bearing iodate by low resolution ESI‐MS would require ^129^I/^127^I ratios on the order of ~10^−3^ to 10^−2^ to produce a distinguishable signal without isotopic or instrumental separation. As a result, natural and most environmental levels of ^129^I are unlikely to be directly observable using the present approach. Instead, ^129^I is best treated as a complementary source tracer, quantified using techniques such as accelerator mass spectrometry, although ESI‐MS can provide complementary constraints on oxygen isotopologue distributions and transformation processes.

Beyond instrumental precision, meaningful clumped isotope measurements require rigorous calibration and correction of instrument‐level effects for quantitative analysis of natural systems. Because clumped isotope measurements (Δ values) are defined relative to a stochastic reference frame, measurements must be anchored to materials of independently constrained isotopic composition. Instrumental effects, including nonlinearity and potential isotope scrambling, must be characterized and corrected through repeated analysis of reference materials.

A critical prerequisite for extending clumped isotope thermometry to IO_3_^−^ is the development of appropriate reference materials. While bulk oxygen isotope measurements are typically anchored to Vienna Standard Mean Ocean Water (VSMOW) and carbonate clumped isotope measurements rely on well‐characterized standards such as ETH‐1 and ETH‐2 [42], no analogous reference materials currently exist for IO_3_^−^ clumped isotopes. Establishing a quantitative IO_3_^−^ clumped isotope framework would require (1) preparation of IO_3_^−^ solutions equilibrated at known temperatures spanning Earth surface, hydrothermal, and nuclear process conditions to define an empirical temperature calibration; (2) production of materials with stochastic isotopologue distributions (Δ = 0) to anchor the reference frame; and (3) interlaboratory comparisons to evaluate reproducibility. Finally, long‐term measurement repeatability must additionally be established through repeated analysis of in‐house IO_3_^−^ standards to quantify external precision achievable on an ESI‐MS platform. Parallel theoretical constraints from ab initio calculations would provide independent estimates of equilibrium clumped isotope values in IO_3_^−^, offering an additional benchmark against which experimental measurements can be evaluated. Together, these analytical and calibration advances define the pathway from the qualitative isotopologue detection demonstrated here toward quantitative clumped isotope thermometry in IO_3_^−^.

The present study therefore demonstrates that ESI‐MS platforms are well suited for experiments involving enriched tracers or process‐driven isotope perturbations. Feasible future experiments could include directly measuring temperature‐ and pH‐dependent oxygen isotope exchange kinetics (described in Section 2.3.3). Further, oxygen isotope tracer experiments could track redox transformations relevant to natural systems or waste simulants.

## 5 Conclusions

This study presents the first exploratory measurements of isotopologue distributions in iodate (IO_3_^−^), demonstrating the feasibility of using ESI‐MS for inorganic oxyanion isotopic analysis. By systematically varying the proportion of H_2_^18^O in spray solvents, we successfully resolved singly substituted (I^16^O_2_^18^O^−^ at *m/z* 177) and clumped isotopologues (I^16^O^18^O_2_^−^ at *m/z* 179 and I^18^O_3_^−^ at *m/z* 181). The relative isotopologue abundances varied systematically with the bulk isotopic composition of the solvent.

The ability to resolve these isotopic distributions establishes ESI‐MS as an accessible, cost‐effective platform for investigating process‐driven isotopic perturbations and enriched tracer experiments in inorganic systems. The thermodynamic framework for IO_3_^−^ clumping grounded in the large reduced‐mass sensitivity of the I–O bond to ^18^O substitution and the rapid oxygen isotope exchange kinetics with water suggests that IO_3_^−^ has potential as a novel paleothermometer and mechanistic process tracer. In Earth sciences, this system could provide new constraints on competing formation pathways within the marine iodine cycle, distinguishing slow equilibrium‐driven oxygen exchange from rapid kinetic oxidation (abiotic photochemical or microbially mediated).

Beyond Earth sciences, the expansion of oxygen and clumped isotope geochemistry into IO_3_^−^ introduces significant opportunities for nuclear forensics and environmental monitoring. The detection of distinctive clumped signatures, particularly those incorporating the fission product ^129^I, could provide new constraints on the thermal and chemical conditions of nuclear fuel reprocessing and support tracking of anthropogenic iodine through complex environmental systems.

Although ESI‐MS is well suited for enriched isotopic studies, extending these measurements to natural abundance clumped isotope thermometry will likely require the enhanced mass resolution and ion counting statistics of high‐resolution platforms, such as Orbitrap‐based mass spectrometers. Future efforts to establish a robust quantitative framework, including the development of iodate reference materials, empirical temperature calibrations, and ab initio predictions of equilibrium isotope distributions, represent critical next steps. Ultimately, this work demonstrates the feasibility of resolving isotopologue distributions in iodate, and potentially other oxyanions, using widely accessible instrumentation. These advances open a new analytical pathway for probing temperature, mechanism, and isotopic history in both natural and anthropogenic systems.

## Author Contributions

**Jamie Lucarelli:** conceptualization, investigation, writing – original draft, writing – review and editing, formal analysis, methodology, visualization. **Amanda R. Bubas:** conceptualization, writing – original draft, investigation, methodology, writing – review and editing, formal analysis, visualization. **Richard M Cox:** conceptualization, funding acquisition, writing – review and editing, methodology, supervision, investigation. **Sophie B. Lehmann:** conceptualization, writing – review and editing. **Aradhna Tripati:** funding acquisition, supervision, writing – review and editing. **Mark Engelmann:** supervision, conceptualization. **Elise R. Conte:** conceptualization, funding acquisition, methodology, supervision, writing – review and editing.

## Supporting information


**Table S1:** Raw intensities for all isotopologues.
**Table S2:** Background corrected intensities for all isotopologues.
**Table S3:** Calculated oxygen isotope equilibration time (*t*_99_) between IO_3_^−^ and water as a function of temperature and pH.

## Data Availability

The data that support the findings of this study are available in the Supporting Information of this article.
